# Angelicin Alleviates Post-Trauma Osteoarthritis Progression by Regulating Macrophage Polarization via STAT3 Signaling Pathway

**DOI:** 10.3389/fphar.2021.669213

**Published:** 2021-06-09

**Authors:** Zhansong Tian, Fanchun Zeng, Chunrong Zhao, Shiwu Dong

**Affiliations:** ^1^ Department of Biomedical Materials Science, School of Biomedical Engineering, Third Military Medical University, Chongqing, China; ^2^ State Key Laboratory of Trauma, Burns and Combined Injury, Third Military Medical University, Chongqing, China

**Keywords:** angelicin, macrophage, polarization, osteoarthritis, inflammation

## Abstract

Post-trauma osteoarthritis (PTOA) is the most common articular disease characterized by degeneration and destruction of articular cartilage (Bultink and Lems, Curr. Rheumatol Rep., 2013, 15, 328). Inflammatory response of local joint tissue induced by trauma is the most critical factor accelerating osteoarthritis (OA) progression (Sharma et al., 2019; Osteoarthritis. Cartilage, 28, 658–668). M1/M2 macrophages polarization and repolarization participates in local inflammation, which plays a major role in the progression of OA (Zhang et al., 2018; Ann. Rheum. Dis., 77, 1524–1534). The regulating effect of macrophage polarization has been reported as a potential therapy to alleviate OA progression. Synovitis induced by polarized macrophages could profoundly affect the chondrocyte and cartilage matrix (Zhang et al., 2018; Ann. Rheum. Dis., 77, 1524–1534). Generally, anti-inflammatory medications widely used in clinical practice have serious side effects. Therefore, we focus on exploring a new therapeutic strategy with fewer side effects to alleviate the synovitis. Angelicin (ANG) is traditional medicine used in various folk medicine. Previous studies have revealed that angelicin has an inhibitory effect on inflammation (Wei et al., 2016; Inflammation, 39, 1876–1882), tumor growth (Li et al., 2016; Oncology reports, 36, 3,504–3,512; Wang et al., 2017; Molecular Medicine Reports, 16, 5441–5449), DNA damage (Li et al., 2019; Exp. Ther. Med., 18, 1899–1906), and virus proliferation (Li et al., 2018; Front. Cell. Infect. Microbiol., 8, 178). But its specific effects on influencing the process of OA were rarely reported. In this study, the molecular mechanism of angelicin *in vivo* and *in vitro* was clearly investigated. Results showed that angelicin could regulate the M1/M2 ratio and function and alleviate the development of PTOA in the meanwhile. Bone marrow monocytes were isolated and induced by macrophage colony-stimulating factor (M-CSF), lipopolysaccharide (LPS) and interferon (IFN)-γ for M1 polarization and interleukin (IL)-4/IL-13 for M2 polarization. Subsequently, repolarization intervention was performed. The results indicate that angelicin can repolarize M1 toward M2 macrophages by upregulating the expression of CD9. Besides, angelicin can also protect and maintain M2 polarization in the presence of LPS/IFN-γ, and subsequently downregulate the expression of inflammatory mediators such as IL-1β and TNF-α. Mechanistically, angelicin can activate the p-STAT3/STAT3 pathway by conducting CD9/gp130 to repolarize toward M2 macrophages. These results suggest angelicin can alleviate the progression of OA by regulating M1/M2 polarization via the STAT3/p-STAT3 pathway. Therefore, angelicin may have a promising application and potential therapeutic value in OA clinical treatment.

## Introduction

Osteoarthritis (OA) is the most common and severe joint disease worldwide and creates a substantial socioeconomic burden, affecting 10% of men and 18% of women over 60 years of age (Glyn-Jones et al., 2015). OA was once considered a complex disease induced by mechanical risk factors (Glyn-Jones et al., 2015; Zhu et al., 2019), but it is thought to be a chronic degenerative disease involving the whole joint rather than so-called wear and tear disease as commonly described (Tu et al., 2019). Both composition changes of cartilage and physical forces can increase the susceptibility to the disruption of the synovial joint (Liu-Bryan, 2015). Among all the pathology-related elements, inflammatory response, as many researchers reported previously, has been verified to play a critical role in the pathogenesis of OA and have a positive correlation with poor prognosis (Bijlsma et al., 2011; Loeser et al., 2016).

Previous studies have shown that OA is a low-grade chronic inflammatory disease, causing damage to multiple articular and periarticular tissues. Emerging evidence suggests local inflammation, synovitis, in particular, plays a crucial role in the pathogenic process of OA (Zhang et al., 2018). Macrophage is a group of heterogeneous cells derived from innate myeloid cells, which profoundly exists in synovial tissue and shapes the inflammatory response of the entire joint. Macrophages were identified to involve multiple physiological and pathological processes (Sharma et al., 2019). The polarization of macrophages could be driven by various cytokines, growth factors and products of microbial metabolism (Veloso et al., 2020). Generally, the classical pathway is the primary activation of polarization of macrophages. Macrophages polarize toward M1 phenotype can be induced by type 1 T helper (Th1) cytokines, including IFN-γ and LPS, upregulating enzyme-inducible nitric oxide synthase (iNOS). M1 phenotype produces reactive oxygen species (ROS) and cytokines such as, IL-1β, IL-6, IL-12, and IL-23 (Rhee, 2016). When chondrocytes are stimulated by IL-1β, cytokines such as prostaglandin E2 (PGE2), which have been proved as a potent mediator of inflammation, are released into the synovial fluid to exacerbate the loss of cartilage (Grieshaber-Bouyer et al., 2019). Meanwhile, the expression of various proteinase such as matrix metalloproteinases (MMPs) is upregulated, resulting in the degradation of articular cartilage. On the contrary, alternative activation of M2 phenotype stimulated by Th2 cytokines including IL-4 or IL-13 upregulates enzyme arginase(Arg)-1, Fizz-1 and Ym-1, increasing the secretion of multiple cytokines such as IL-4, IL-10, IL-13, and transforming growth factors (TGF)-β to participate in the tissue recovery conditions and reduce the inflammatory response (Mantovani et al., 2002). Several signal pathways, such as signal transducer and activator of transcription (STAT) family have been implicated in involving inflammation and macrophage polarization (Zhou et al., 2020). CD9/gp130/STAT3 signal pathway activation has been proved to participate in repolarization (Gupta et al., 2018). Phosphorylation of downstream transcription factor STAT3 leads to activation of IL-6 signal pathway, initiating repolarization and the maintenance of M2 macrophages. Moreover, the IL-6 receptor, gp130, is stabilized by the CD9 molecule to activate the phosphorylation of downstream STAT3. The balance of macrophage polarization is tightly linked with the duration of repair and the modulation of immune system.

Breaking the original balance of macrophage polarization has been proved to contribute to accelerate the pathological process of various diseases. Exorbitant activation of the M1 type phenotype can cause tissue damage and metabolism disorders (Xie et al., 2020; Yang et al., 2020). Increased inflammatory macrophages have been widely reported positively related to diabetes (Huangfu et al., 2020). Also, infiltration of M1 macrophages in adipose tissue reflects local tissue inflammation (Braune et al., 2017). The ratio of M1 to M2 is positively correlated with nephritis and other chronic inflammation. M2 macrophage is also related to the tissue repair and pathological scar formation (Xu et al., 2020). Functionally, M2 macrophages are crucial for effective suppression and clearance of local infections and inflammation caused by M1 macrophage and its pro-inflammatory cytokines. Meanwhile, M2 macrophage also show its benefit for tissue remodeling, angiogenesis, and extracellular matrix formation (Mapp and Walsh, 2012). Therefore, M1/M2 macrophages can be recognized as a great potential target in healing damaged tissue and treating chronic diseases requiring long-term therapies. Regulating the ratio of M1/M2 macrophages can be considered safe and effective OA therapeutic strategies (Zhang et al., 2018).

Angelica Sinensis, used in traditional Chinese medicine, is widely recognized as an effective herb to heal multiple diseases. Angelica is gaining ground all over the world, including Europe, North American and Southeast Asia. In folk medicine, angelica is believed as an anti-inflammatory, anti-aging medicine with wide use (Wei et al., 2016; Ge et al., 2019). Angelicin is isolated from Angelica Sinensis considered as an anti-frostbite, analgesia, hemostasis active ingredients. In recent years, angelicin has been reported as anti-inflammation, anti-bacterial, anti-tumor, immunomodulatory, pro-apoptotic and anti-virus activities (Li et al., 2016; Wang et al., 2017). Studies have shown that angelicin has a strong immunomodulatory effect (Lampronti et al., 2017). However, the underlying molecular mechanism and a potential target for treatment remain unknown.

In this study, for the very first time, we revealed that angelicin is an effective pharmaceutical therapy for regulating M1/M2 macrophage polarization. Angelicin plays a role in suppressing M2 polarization, especially when M2 macrophages repolarized toward M1 and facilitating M2 polarization through the STAT3 pathway. These findings have implied that angelicin may have potential therapeutic value in the future to alleviate the inflammation of articular and periarticular tissue and then restrain the progression of OA.

## Materials and Methods

### Reagents and Antibodies

Angelicin with purity > 95% was purchased from Med Chem Express (Monmouth Junction, NJ, United States) and dissolved in DMSO. Alpha Minimum Eagle’s Medium (alpha-MEM), penicillin (10,000 units/ml), streptomycin (10,000 mg/ml) and fetal bovine serum (FBS) were purchased from Hyclone (Waltham, MA, United States). Soluble mouse recombinant M-CSF was purchased obtained from R&D system (Minneapolis, MN, United States). Interleukin (IL)-4, IL-13, LPS, and IFN-γ were purchased from PeproTech Inc. (Rocky Hill, NJ, United States). The Cell Counting Kit-8 (CCK-8) was purchased from Med Chem Express (Monmouth Junction, NJ, United States). Anti-CD9, anti-gp130, anti-STAT3, anti-phospho-STAT3 (Ser727), anti-CD206, anti-iNOS, anti-Arg-1, anti-β-actin antibodies were purchased from Bioworld Technology (St. Louis Park, MN, United States).

### Cell Proliferation Assay Cell Counting Kit-8

The cytotoxicity evaluation of angelicin in bone marrow monocytes was examined using CCK-8 kit. BMMs isolated from mice femur were seeded in 96-well plates at a density of 3 × 10^3^ per well and were treated with a range of 10 μmol/L-100 µmol/L for 24 or 48 h. Next, CCK-8 kit solution was added, and then the system described above was incubated for 2 h away from light. Absorbance was detected at 450 nm and recorded by a microplate absorbance reader (Bio-Tek, Elx800, United States).

### Cell Culture and Incubation

BMMs were isolated from the femurs of 6-week-old C57BL/6 mice under aseptic condition. α-MEM with 10% FBS was applied for flushing the bone marrow capacity. The mixtures described above were filtered with 200 mesh nylon filter. BMMs were seeded into 60mm cell culture dish containing a complete medium composed of 90% α-MEM and 10% FBS in an incubator set at 37 C with 5% CO_2_. BMMs were incubated for 72 h in α-MEM supplemented with 30 ng/ml M-CSF, 10% FBS, 100 units/ml penicillin and 100 mg/ml streptomycin. Then, non-adherent cells were removed with PBS. After reaching the confluence of 80%, BMMs were seeded in 6-well plate with the complete medium in an incubator at 37 C with 5% CO_2_.

### RNA Isolation and Polymerase Chain Reaction

Quantitative real-time polymerase chain reaction (qRT-PCR) was used to investigate marker gene expression in M1/M2 macrophages in different groups. BMMs were seeded in 6-well plates at a density of 1 × 10^5^ cells per well and cultured in a complete medium containing 30 ng/ml M-CSF for 24 h, with 20 ng/ml IL-4 and 20 ng/ml IL-13 or 20 ng/ml LPS and 100 ng/ml IFN-γ for 36 h. Then cells were lysed using Trizol reagent (Invitrogen) 1 ml per well. Complementary DNA (cDNA) was synthesized from 1μg RNA from each sample using a reverse transcriptase kit (Takara, Japan). For real-time PCR, 1ug RNA was mixed with primers and SYBR green super mix. The gene expression levels were calculated with 2 ^–∆∆CT^ values by CFX Manager™ Software v3.1(Bio-Rad). Glyceraldehyde 3-phosphate dehydrogenase (GAPDH) was used as a control gene in this experiment. The primer sequences are listed in Table 1.

**TABLE 1:** Primer sequence used in qRT-PCR experiment.

Gene	Sequence
INOS	Forward: 5′-ACA​TCA​GGT​CGG​CCA​TCA​CT-3′
	Reverse: 5′-CGT​ACC​GGA​TGA​GCT​GTG​AAT​T-3′
Arg-1	Forward: 5′-AGG​ACA​GCC​TCG​AGG​AGG​GG-3′
	Reverse: 5′-TGG​ACC​TCT​GCC​ACC​ACA​CC-3′
GAPDH	Forward: 5′-CTA​AGG​CCA​ACC​GTG​AAA​AG-3′
	Reverse: 5′-ACC​AGA​GGC​ATA​CAG​GGA​CA-3′
IL-1β	Forward: 5′-GAA​ATG​CCA​CCT​TTT​GAC​AGT​G-3′
	Reverse: 5′-TGG​ATG​CTC​TCA​TCA​GGA​CAG-3′
IL-6	Forward: 5′-CTG​CAA​GAG​ACT​TCC​ATC​CAG-3′
	Reverse: 5′-AGT​GGT​ATA​GAC​AGG​TCT​GTT​GG-3′
TNF-α	Forward: 5′-CAG​GCG​GTG​CCT​ATG​TCT​C-3′
	Reverse: 5′-CGA​TCA​CCC​CGA​AGT​TCA​GTA​G-3′

### Western Blotting

Cell samples were harvest at 36 h in 6-well plates after the intervention described above. Cells were lysed in RIPA lysis buffer with phenylmethanesulfonyl fluoride (PMSF). Protein samples were loaded to SDS-PAGE with 20 ul per lane and then transferred to PVDF membranes. Next, membranes were blocked with 5% bovine serum albumin (BSA) blocking agent for 1.5 h and washed with TBS-T buffer for three times, 15 min for each. Primary antibodies against iNOS (1:1,000), Arg-1 (1:1,000), CD9 (1:1,000), gp130 (1:1,000), STAT3 (1:500), p-STAT3 (1:500), β-actin (1:2,000) were incubated with the membranes in the BSA solution overnight at 4 C. Subsequently, the membranes were incubated with horseradish peroxidase (HRP)-conjugated goat anti-rabbit secondary antibodies (1:1,000) for 1.5 h and washed three times with TBST buffer. Lastly, the Chemi Doc XRS + Imaging System (Bio-Rad, United States) was performed to capture images of the protein bands, which were analyzed with the ImageJ v2.0. β-actin was used as an internal control in this experiment.

### Enzyme-Linked Immunosorbent Assay

The secretion of IL-1β and TNF-α of polarized macrophages in cell supernatant was measured by ELISA Kits (Cloud clone, China) according to the instructions. All measurements were repeated five times.

### Mice

C57BL/6 mice were obtained from the animal center of the Third Medical University (Chongqing, China). All experiments in this research were performed according to the Third Military Medical University Sciences Guide for Laboratory Animals. Animal suffering weas reduced by all means with all our forces. All animals were maintained at 25 C under 12 h’ light/dark cycle with 50% humidity. Eighteen 6–8 weeks of female C57/BL6 mice were separated randomly into three equal groups: Control, LPS and LPS + ANG groups. The Control group were performed intraperitoneal injection with Saline. LPS group was performed intraperitoneal injection with LPS (2 mg/kg, body weight). LPS + ANG group were performed intraperitoneal injection with LPS (2 mg/kg, body weight) and angelicin (20 mg/kg, body weight). Injections were performed intraperitoneal every other day continuously for two weeks. 14 days later, all mice were sacrificed, and the femur was isolated under aseptic condition. Femurs were fixed with 4% paraformaldehyde for 48 h and decalcified in EDTA (pH = 7.2) for 14 days for further testing.

To confirm the protective effect of angelicin, DMM models were established as previously described (Chen et al., 2021). According to the previous studies protocol, the induced DMM model is similar to the pathological process of human joint damage. After anesthetization, a 1 cm incision was made on the medial side of the joint to fully expose the joint capsule and transect the medial meniscotibial ligament. The joint capsule and skin were subsequently sutured by absorbed sutures. Mice in the sham surgery group performed the same incision with ligaments kept. After surgery, fifteen mice were randomly divided into three groups: Sham group, DMM group and DMM + ANG group. Mice were performed an intraperitoneal injection of 20 mg/kg angelicin or saline once a day for 8 weeks after surgery.

### Flow Cytometry

Flow cytometry was applied for examining CD206^+^/F4/80^+^and iNOS^+^/F4/80^+^ cell proportion of macrophages. After complete euthanization of mice, macrophages were isolated from femurs and resuspended in ice-cold PBS. Cells seeded in the 6-well plate were digested into single cells by trypsin. Half of the collected cells were incubated with PE-conjugated CD206 and AF 780-conjugated iNOS antibodies (Thermo Fisher Science, United States) for 45 min, respectively. Cells were further incubated with BV421-conjugated F4/80 antibody (Thermo Fisher Science, United States) for 45 min at 4°C. iNOS^+^F4/80^+^ macrophage was recognized as M1 phenotype and CD206^+^F4/80^+^ macrophage was recognized as M2 phenotype.

For apoptosis detection, Annexin-V Apoptosis Detection Kit (Beyotime, China) was used to detect apoptosis of BMMs. Cells were harvested with 0.25% trypsin and washed with PBS. Then cells were resuspended with 100 μL Annexin-V and propidium iodide (PI) staining buffer diluted in PBS for 20 min on the ice from light. The cells were analyzed on CytoFLEX Flow Cytometer (Beckman, IN, United States), and data were analyzed in FlowJo (Ashland, OR, United States) followed by instructions.

### Immunofluorescence

To further investigate the polarization and repolarization of macrophages, isolated BMMs were stimulated with LPS/IFN-γ for M1 polarization and IL-4/IL-13 for M2 polarization alone or combined with 30 μmol/L angelicin. After treatment, the samples were rinsed with PBS for three times. Then the samples were fixed by 4% paraformaldehyde and permeabilized by Triton X-100 diluted in PBS for 10 min. Samples were blocked with 5% BSA at 37°C for 30 min and washed with PBS, and incubated with iNOS (1:150) and CD206 (1:150) primary antibodies overnight at 4°C. Subsequently, samples were washed by PBS for three times and incubated with AF647-conjugated secondary antibody (1:400) for 1 h and stained with DAPI for 5 min at room temperature. After staining, the fluorescence microscope (Olympus Inc., Tokyo, Japan) was used to observe the plates and slides. Fluorescence intensity was measured and quantified using ImageJ v2.0.

### Histopathologic Analysis

The keen joints were isolated on the day of execution (8 weeks after surgery) and fixed in 4% paraformaldehyde for 48 h and decalcified in EDTA (pH = 7.2) for 14 days. After dehydration, embedding and cutting, the slides were stained with Safranin-O/Fast Green and hematoxylin and eosin (H&E). An experienced histological investigator blinded to evaluate medial femoral condyle and medial tibial plateau according to the International Osteoarthritis Research Society International (OASRI) scoring system as described previously (Chen et al., 2021).

### Statistical Analysis

All values are representative of at least three parallel experiments of resembling results except independently demonstrated. Data are expressed as mean ± SD and comply with the normal distribution. Independent groups are compared by one-way ANOVA followed by Student-Newman-keul post hoc tests to determine the significant difference between results, with *p < 0.05 and **p < 0.01 being regarded as significant.

## Results

### Cytotoxicity Evaluation of Angelicin on Bone Marrow Monocytes

**FIGURE 1:**
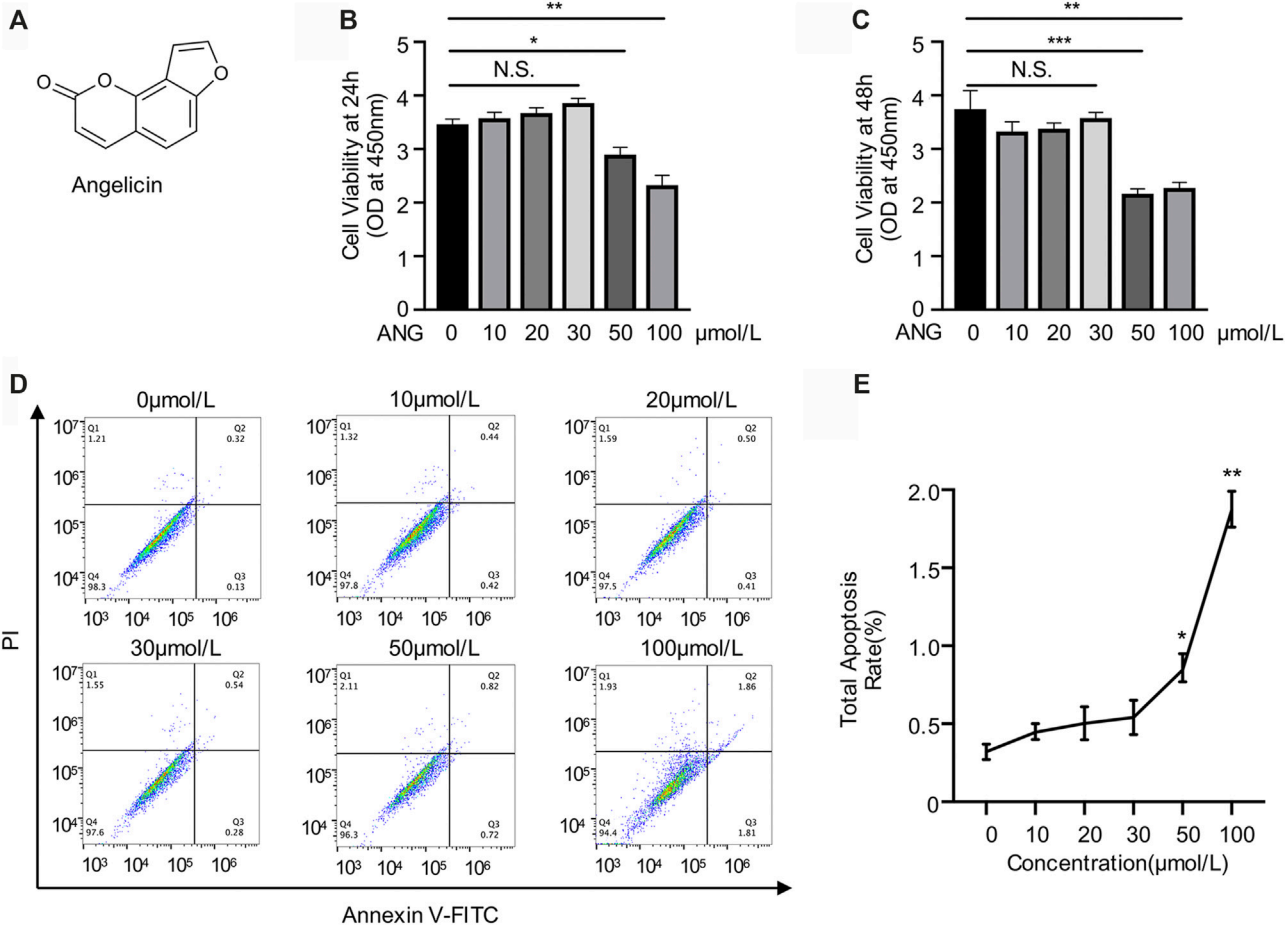
Evaluation of angelicin toxicity in bone marrow macrophages (BMMs). **(A)** Chemical structure of angelicin. **(B, C)** Effect of angelicin on the cell viability of BMM. The cytotoxic effects of angelicin were detected at the different concentrations for 24 and 48 h using CCK-8 assay. **(D, E)** Representative image of apoptosis rate detected by flow cytometry of each group. The values presented are the mean ± SD of five independent experiments. *p < 0.05, **p < 0.01, ***p < 0.001 (ANOVA).

Angelicin is a kind of furocoumarin, a naturally occurring tricyclic aromatic compound. The chemical formula of angelicin is showed in the figure (Figure 1A). To detect the cytotoxicity of angelicin, BMMs were isolated and treated with various concentrations of angelicin for 24 and 48 h followed by CCK-8 assays. The results revealed that low concentration of angelicin (≤ 30 µmol/L) had no toxic effect on the proliferation of BMMs (Figures 1B,C). Apoptosis assays were conducted using flow cytometry, and results were consistent with CCK-8 (Figures 1D,E). Our results showed that angelicin has a mild toxic reaction at high concentration. Based on the results of the toxicity evaluation, the dose of angelicin 30 µmol/L was chosen for further experiments.

### M1 Macrophage is Repolarized in the Presence of Lipopolysaccharide/Interferon-γ and M2 Polarization is Protected by Angelicin *in vitro*

**FIGURE 2:**
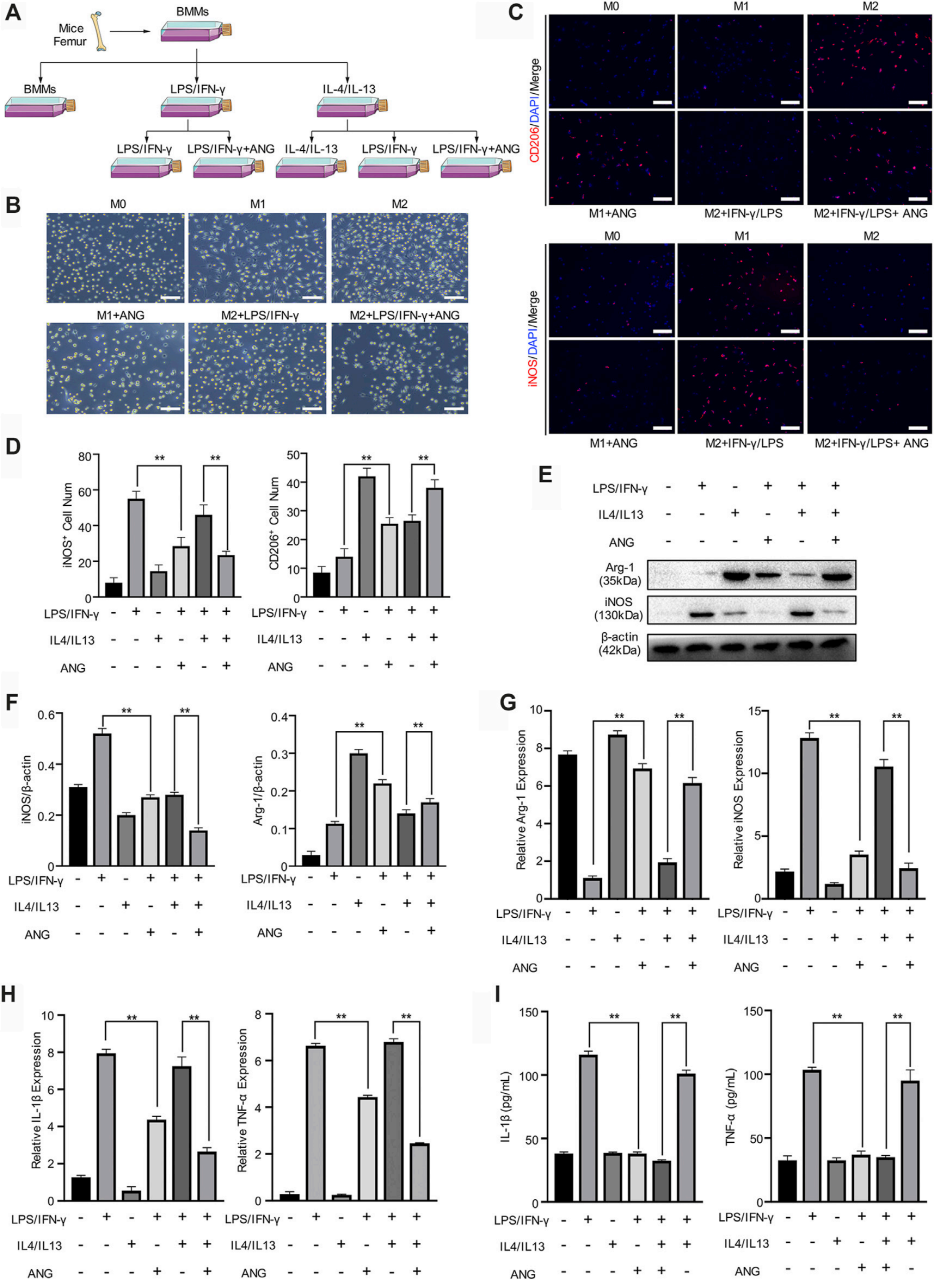
Angelicin protectsM1 polarization and M2 macrophage polarization *in vitro*. **(A)** Schematic diagram of bone marrow macrophages (BMMs) isolation and macrophage M1/M2 polarization and repolarization strategy. **(B)** Representative morphology images in each group of three independent experiments. Bar represents 10 μm. **(C, D)** Immunostaining of enzyme-inducible nitric oxide synthase (iNOS) and CD206 (red) in isolated BMMs in different groups with quantification of iNOS^+^ and CD206^+^ cell numbers. **(E, F)** Western blot assay and qRT-PCR assay of Arg-1, iNOS and β-actin in BMMs. **(G)** qRT-PCR assay of IL-1β and TNF-α expression in BMMs. **(H)** ELISA for IL-1β and TNF-α concentration from cellular supernatant. The data in figures represents the mean ± SD. Significant differences are indicated as **p < 0.01. Scale bar represents 30 μm. DAPI, 4′,6-diamidino-2-phenylindole; IL, interleukin; LPS/IFN-γ, lipopolysaccharide/interferon-γ; ANG, angelicin.

To evaluate the effect of angelicin on macrophage polarization *in vitro*, primary BMMs were isolated for further investigation. BMMs were stimulated with LPS/IFN-γ for M1 polarization and IL-4/IL-13 for M2 polarization. During the stimulation of M1 macrophage, angelicin treatment has shown the effect of angelicin on this polarization procedure. However, the repolarization procedure was performed that M2 macrophages were treated with LPS/IFN-γ alone or combined with angelicin was conducted to observe repolarization (Figure 2A). Besides, given that M1 and M2 macrophages have distinct differences in morphology, we captured the microscope images of each experiment group and found that the microscopic morphology of the cells also changed (Figure 2B). Interestingly, we found that angelicin could produce an inhibitory effect on the polarization from M0 macrophages to M1 macrophages with decreased expression of iNOS and slightly changed expression of CD206. M2 macrophages treated with LPS/IFN-γ have shown robust repolarization toward M1 phenotype; However, with the presence of angelicin, LPS/IFN-γ failed to repolarize M2 phenotype toward M1 macrophages (Figure 2C). Quantification analysis of CD206^+^ (represent as M2 macrophages) cell number and iNOS^+^ (represent as M1 macrophages) cell number was shown on the left (Figure 2D). To ascertain the changes, we further examined the expression of iNOS and Arg-1 on the protein level. Western blot results implied that M1 macrophages treated with angelicin and M2 macrophages showed higher expression of Arg-1 and lower expression of iNOS. Moreover, M2 macrophages treated with both LPS/IFN-γ and angelicin showed fewer iNOS expression compared with the M2 macrophages treated with LPS/IFN-γ (Figures 2E,F). Coincidently, qRT-PCR results showed that the expression of Arg-1 was significantly higher in M2 macrophages and M2 macrophages treated with LPS/IFN-γ and angelicin, while the expression of iNOS was markedly higher in M1 macrophages and M2 macrophages treated with LPS/IFN-γ (Figure 2G). Not surprisingly, the relative expression and secretion level of inflammatory factors including IL-1β and TNF-α decreased (Figures 2H,I). All the results mentioned above suggested that angelicin suppressed the polarization of M0 macrophages toward M1 macrophages and the expression of inflammatory factors. Meanwhile, it could also dramatically suppress the repolarization of M2 macrophages toward M1 phenotype.

### Angelicin Modulates M1/M2 Polarization via STAT3 Signal Pathway and Decreases the Secretion of Inflammatory Factors

**FIGURE 3:**
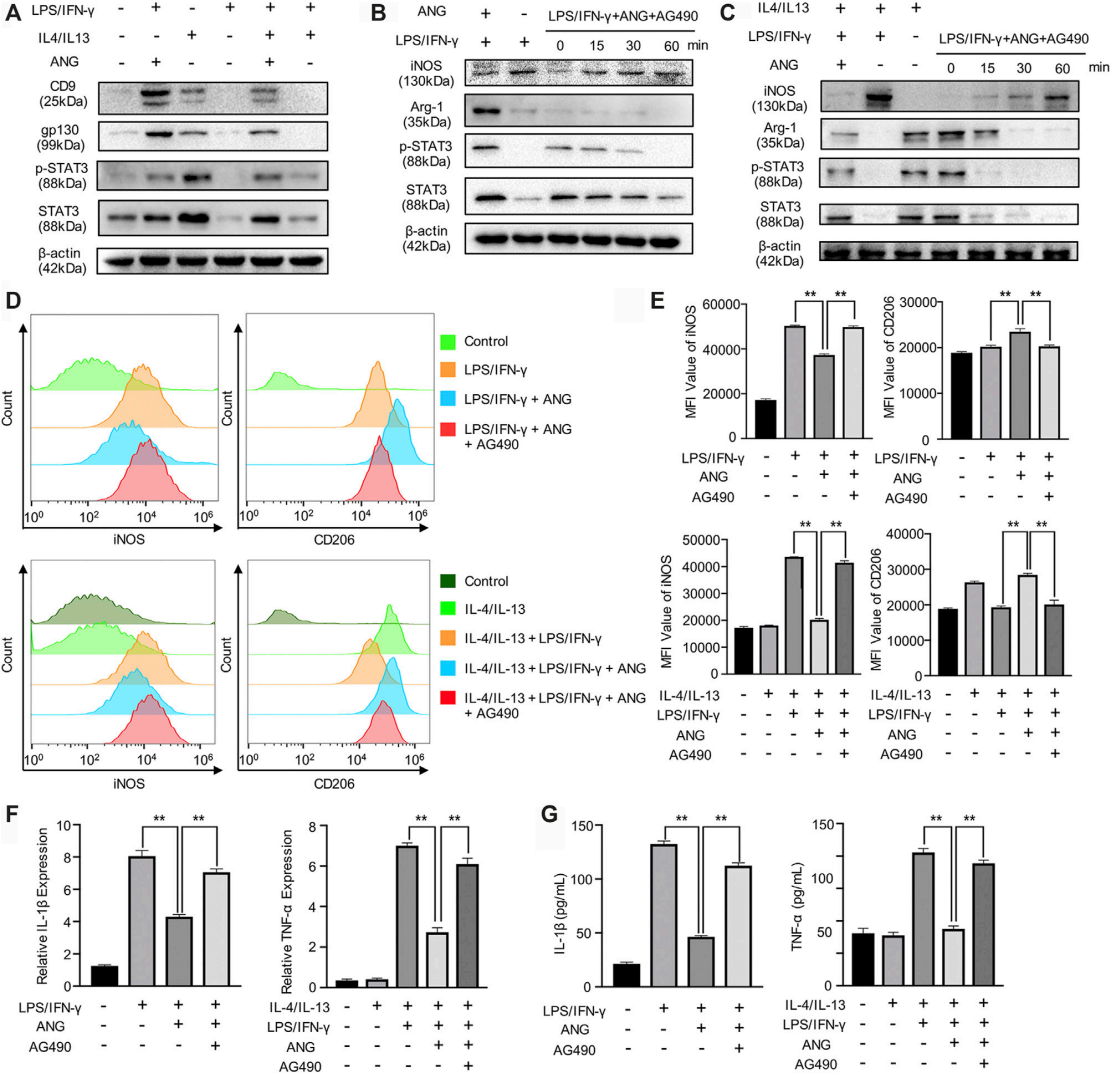
Angelicin could regulate M1/M2 polarization via STAT3 signal pathway. **(A)** Western blot analysis of CD9, gp130, STAT3, p-STAT3, and β-actin in BMMs treated with LPS, IL-4/IL-13, LPS + Angelicin, LPS + IL-4/IL-13 + Angelicin or LPS + IL-4/IL-13. **(B)** Western blot analysis of iNOS, Arg-1, STAT3, p-STAT3, and β-actin in BMMs treated with LPS, LPS + Angelicin, LPS + IL-4/IL-13 + Angelicin + AG490. **(C)** Western blot analysis of iNOS, Arg-1, STAT3, p-STAT3, and β-actin in M2 macrophages treated with LPS, LPS + Angelicin, LPS + IL-4/IL-13 + Angelicin + AG490. **(D, E)** Flow cytometry analysis of iNOS and CD206 expression of BMMs. **(F)** qRT-PCR assay of IL-1β and TNF-α expression in BMMs. **(G)** ELISA for IL-1β and TNF-α concentration from cellular supernatant.

To investigate the potential mechanism, we focused on the effects of angelicin on the p-STAT3/STAT3 pathway, which is necessary for M1 polarization. We first examined the impact of angelicin on the repolarization of M1 macrophage. BMMs were treated with LPS/IFN-γ to inhibit the STAT3 signal pathway. Western blot analysis was performed to detect the protein level of p-STAT3 and STAT3. The results showed that LPS/IFN-γ could suppress the activation of p-STAT3 and STAT3 in total protein. We then detected the expression of CD9 and gp130, which is necessary for activating the STAT3 signaling pathway. The results showed that angelicin treatment could increase the expression of CD9/gp130 and phosphorylation of STAT3 (Figure 3A). On the contrary, AG-490, which could specifically inhibit phosphorylation of STAT3, was capable of diminishing the protective effect of angelicin overtime during the process of M0 macrophage polarization toward M1 cells (Figure 3B) or M2 macrophage repolarization toward M1 cells (Figure 3C). Western blot results of Figure 3 were quantified and showed in supplementary data (Supplementary Figure S1). Flow cytometry results showed that angelicin could maintain the M2 phenotype and ward off repolarization with the presence of LPS and IFN-γ while AG-490 could counter its effect (Figure 3D). Mean fluorescence intensity (MFI) was measured using Flow jo and listed on the right (Figure 3E). In accordance with previous results, qRT-PCR results showed that AG-490 could partially reverse the lower expression of IL-1β and TNF-α (Figure 3F). ELISA assay was also conducted to measure the secretion of inflammatory factors to investigate the immunomodulatory effect of angelicin and impact of AG-490. The secretion of IL-1β and TNF-α decreased after angelicin treatment as expected, while AG-490 could neutralize the protective effect of angelicin (Figure 3G). Above all, these results revealed that angelicin could regulate M1/M2 polarization via CD9/gp130/STAT3 signaling pathway and decrease the secretion of inflammatory factors.

### Angelicin Robustly Inhibits Lipopolysaccharide Induced M1 Polarization *in vivo*

**FIGURE 4:**
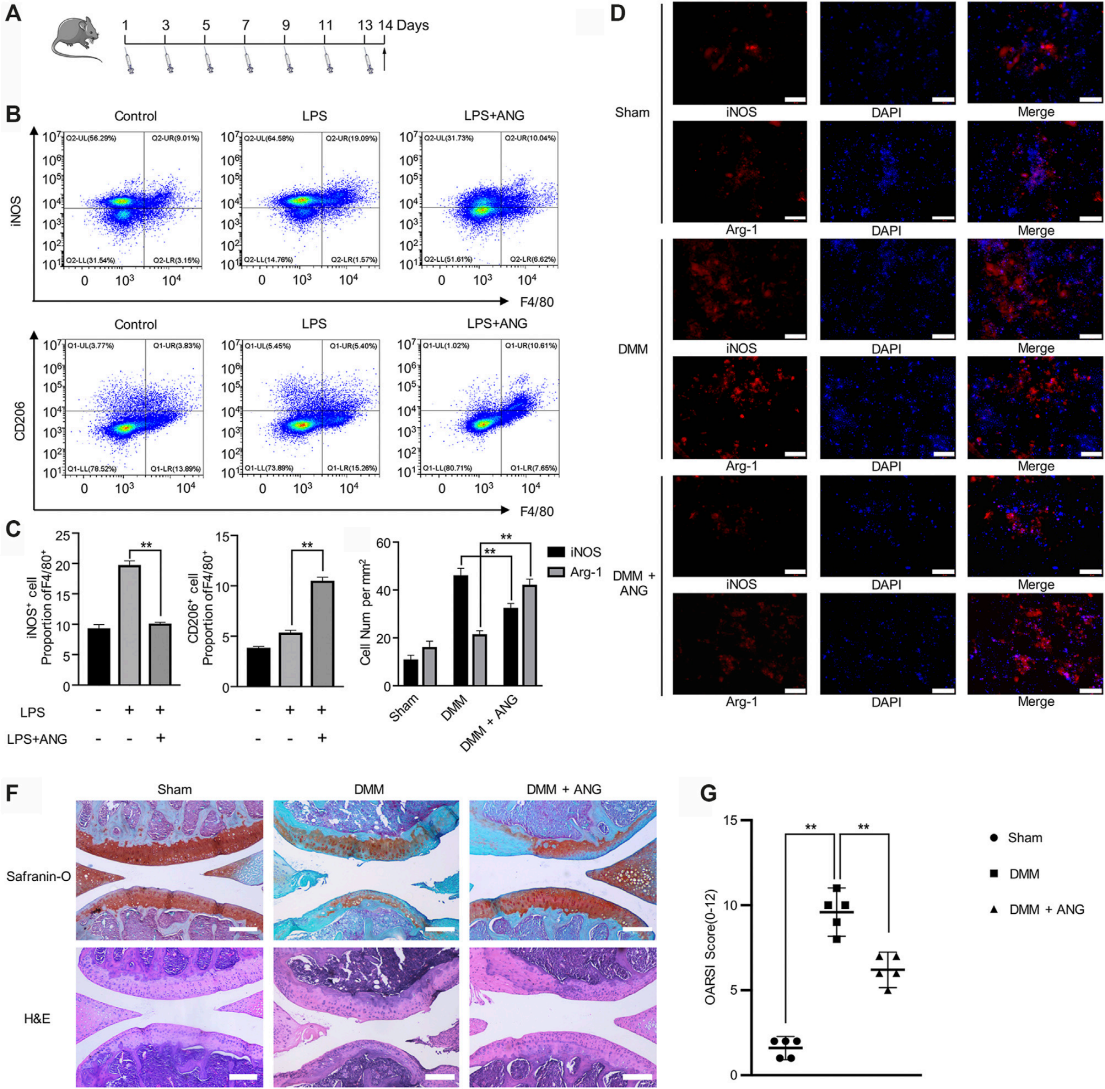
Angelicin alleviates PTOA development by regulating M1/M2 polarization. **(A)** Experiment design of treatment on LPS-induced mice. **(B, C)** Flow cytometry analysis of iNOS^+^F4/80^+^ and CD206^+^F4/80^+^macrophage in total bone marrow cells. **(D, E)** Immunostaining of iNOS and CD206 (red) in DMM model. Bar represents 200 μm. **(F)** Representative Safranin-O staining and H&E staining of cartilage and articular tissues from the different experimental groups (scale bar = 50 µm). **(G)** Diagram showing the OARSI scores of the articular cartilage.

In order to verify the regulatory effects of angelicin on macrophage polarization *in vivo*, LPS induced inflammatory mouse model was performed. Intraperitoneal injection of LPS was given every other day for two weeks (Figure 4A). iNOS^+^ and F4/80^+^ double-positive macrophages were considered as M1 macrophages, while CD206^+^ and F4/80^+^ double-positive macrophages were considered as M2 macrophages. The flow cytometry results indicated that the macrophages from mice bone marrow showed a significant increase of M1 macrophage percentage in LPS-treated mice compared with PBS-treated mice (Figure 4B). Also, the percentage of M2 macrophage significantly reduced in LPS-treated mice compared with M1 macrophage percentage (Figure 4B). However, after angelicin treatment, the increased percentage of M1 phenotype macrophage with the presence of LPS could be reduced, while the percentage of M2 phenotype macrophage increased (Figures 4B,C). Results showed that M1/M2 ratio was significantly reduced in LPS-treatment group after angelicin treatment.

### Angelicin Alleviates Osteoarthritis Development in DMM Mouse Model

In DMM mouse models, immunofluorescence results showed that the number of M2 macrophages decreased in synovial tissue while M1 macrophages did oppositely. However, angelicin treatment could robustly restore M1/M2 ratio (Figures 4D,E). Histological analysis of changes in joint structure and cartilage matrix was performed through H&E staining and Safranin-O/fast green staining, respectively. The sham group showed normal cartilage surface and positive red stain. The DMM group showed degradative cartilage surface and disorganized subchondral structure. The angelicin treatment group showed ameliorated cartilage surface compared with the DMM group (Figure 4F). The OARSI score of the DMM group was higher compared with the sham group (Sham vs. DMM, *p* < 0.01), while the angelicin treatment group was lower compared with the DMM group (DMM vs. DMM + ANG, *p* < 0.01) (Figure 4G).

## Discussion

It is widely reported that OA is the most common and disabling type of arthritis, causing heavy health burdens with severe complications for the affected individuals (Walsh et al., 2007). At present, the prevalent therapy of OA primarily aims to relieve the symptoms, such as continuous pain and cartilage degradation (Bijlsma et al., 2011). However, long-term intake of the drugs like Non-Steroidal Anti-inflammatory Drugs (NSAIDs) would cause serious side effects such as peptic ulcers, kidney damage, and cardiovascular disease (Chu et al., 2019). Therefore, more and more emphasis has been placed on the attempt to alleviate the OA-related symptoms and reduce the side effects induced by drug treatment. Hence, plant extracts have drawn attention of researchers in recent years.

Previous studies suggest that angelicin was potentially advantageous in preventing inflammatory diseases. Angelicin, an ancient herb widely used worldwide, was recently reported to have various effects on regulating inflammatory response (Wei et al., 2016). In this study, we figure out how angelicin exerts influence on macrophages polarization on the cellular and molecular level. As results show, angelicin can regulate macrophage polarization via CD9/gp130/STAT3 pathway. We found that angelicin could facilitate M1 phenotype macrophages activated by LPS/IFN-γ to M2 phenotype macrophages. In terms of molecular mechanisms, M2 phenotype macrophages were protected by angelicin in the presence of LPS/IFN-γ via enhanced phosphorylation of STAT3.

M1/M2 macrophages have been formulated decades ago to be a group of cells with high plasticity and polarization under the conditions of multiple cytokines (Lu and Chen, 2019). Macrophages polarization is a special process in response to the change of microenvironment stimuli and signals. M1 and M2 macrophages have completed different expression profiles of cytokines. M1 macrophages have a higher expression of pro-inflammatory cytokines, including TNF-α, IL-1β, IL-6, IL-12, IL-23, and cyclooxygenase-2 (COX-2) induced by LPS/IFN-γ, therefore endowed with robust anti-inflammation and anti-tumoral function (Davies and Taylor, 2015). While, ROS, one of the products of M1 macrophages is responsible for tissue damage, impairing tissue regeneration caused by excessive activation of M1 macrophages. M2 macrophages were polarization by Th2 cytokines such as IL4/IL-13 via activating STAT pathway through interleukin receptor (ILR), thereby upregulating Arg-1, C-C Motif Chemokine Ligand (CCL) 17, CCL 24 (Wynn and Vannella, 2016). The duration of M2 macrophages activation can mediate lung eosinophilia and airway inflammation. Moreover, the polarization of macrophages is quite common in the resistance of infection and regeneration of tissue damage. Angelicin is capable of regulating M1 macrophages to M2 phenotype. M1 macrophages were induced by Th1 cytokines like LPS and IFN-γ, which is regarded as the most recognized method. In contrast, M2 macrophages were induced by IL-4 and IL-13. As reported previously, the STAT pathway was activated, STAT1 and STAT6 in particular (Ding et al., 2019). Also, synovial tissue inflammation induced by polarized macrophages have been verified to facilitate the degradation of cartilage matrix (Wang et al., 2019).

Angelicin have been reported for their special cellular biological functions these years. Recent research has shown that angelicin can inhibit the malignant behavior of human cervical cancer. Combined administration of psoralen and angelicin can significantly regulate anti-inflammation and osteogenesis effect (Li et al., 2018). Meanwhile, angelicin can also show its antiviral activity against γ-herpesvirus (Cho et al., 2013). One of our recent researches showed that M1/M2 ratio alteration also facilitates estrogen-deficiency-induced osteoporosis (Dou et al., 2018). Angelicin-mediated repolarization will be a potential target to treat chronic inflammation.

**FIGURE 5:**
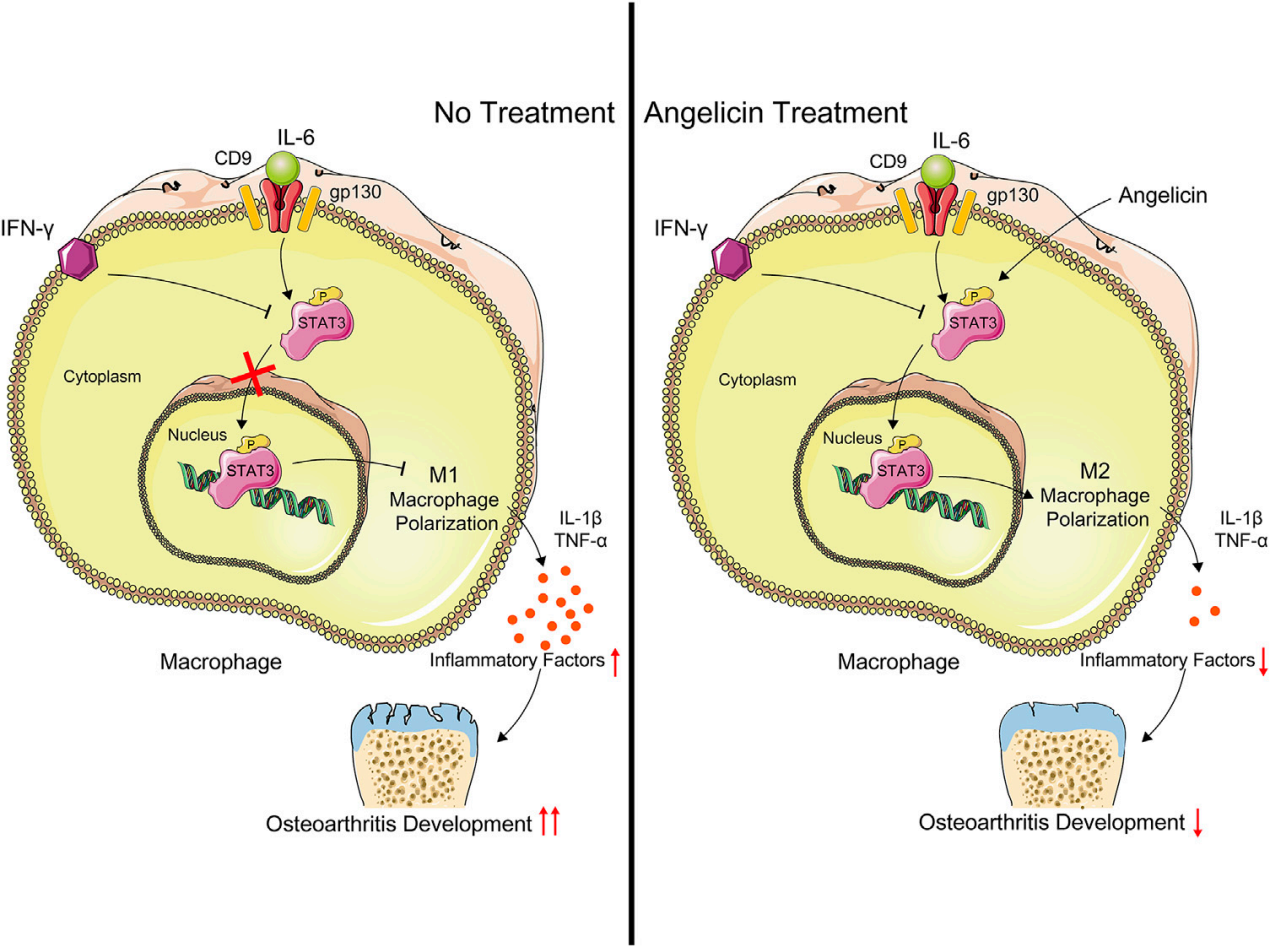
Schematic illustration of the potential protective effects of angelicin in osteoarthritis development. Angelicin increased the phosphorylation of STAT3 and activated M2 macrophage polarization with the presence of LPS and IFN-γ.

In the study, we evaluated the effect of angelicin in regulating the polarization of macrophages both *in vivo* and *in vitro*. Our results had shown that angelicin could inhibit M1 polarization and promote M2 polarization in synovial tissues. Angelicin is protective of maintaining the M2 phenotype in the presence of IFN-γ via CD9/gp130/STAT3 pathway to alleviate synovitis in OA. Angelicin can significantly increase the expression of STAT3 and phosphorylation of STAT3 and then facilitate the expression of M2-related genes (Figure 5). In animal experiments, the angelicin treatment group showed smooth cartilage surface and integrated cartilage matrix, which is similar to healthy cartilage. Also, angelicin has been widely used in clinical practice. To some extent, our study revealed molecular mechanism of angelicin against PTOA. However, to further evaluate the clinical application value of angelicin, pharmacokinetic study and clinical study are still much-needed.

Overall, our studies suggest that angelicin could regulate M1/M2 ratio via CD9/gp130/STAT3 pathway to reduce the inflammatory response and then protect cartilage in OA. These results further raise the possibility that angelicin could be a potential therapeutic agent against OA.

## Data Availability

The original contributions presented in the study are included in the article/Supplementary Material, further inquiries can be directed to the corresponding author.
