# Supplementary figures and images for: Angelicin Alleviates Post-Trauma Osteoarthritis Progression by Regulating Macrophage Polarization via STAT3 Signaling Pathway
Source: Front Pharmacol. 2021 Jun 9;12:669213. doi: 10.3389/fphar.2021.669213 (PMC8223070; doi:10.3389/fphar.2021.669213)

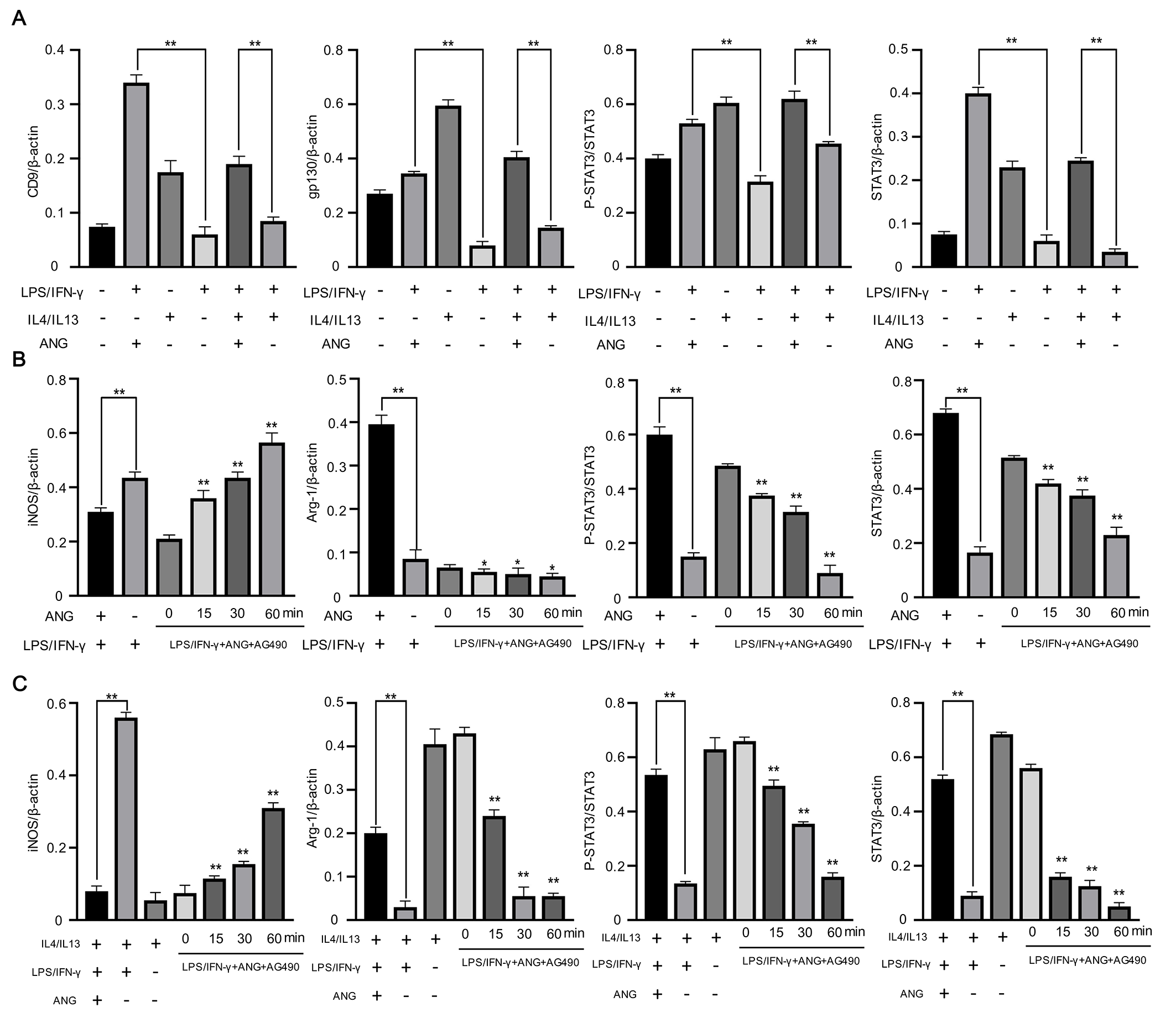

Supplement: Supplementary file 2 [file Image1.TIF]
